# Combining frontal tDCS with walking rehabilitation to enhance mobility and cognition: a pilot clinical trial

**DOI:** 10.1093/geroni/igaa057.1577

**Published:** 2020-12-16

**Authors:** David Clark, Sudeshna Chatterjee, Jared Skinner, Paige Lysne, Samuel Wu, Dorian Rose, Adam Woods

**Affiliations:** 1 University of Florida, Gainesville, Florida, United States; 2 Appalachian State University, Boone, North Carolina, United States; 3 Aging and Geriatric Research, Gainesville, Florida, United States; 4 Department of Biostatistics, Gainesville, Florida, United States; 5 Clinical and Health Psychology, Gainesville, Florida, United States

## Abstract

Walking function is compromised with older age, particularly for cognitively demanding complex walking tasks. Frontal lobe brain networks are important to both complex walking and cognitive function. There is a need for interventions that target this brain region. This pilot study assessed a novel intervention to enhance both walking and executive function in older adults. The primary hypothesis was that eighteen sessions of frontal lobe tDCS combined with complex walking rehabilitation would be feasible, safe, and show preliminary efficacy for improvements in walking and cognition. Eighteen participants were randomized to one of three intervention groups: active tDCS and rehabilitation with complex walking tasks (Active/Complex); sham tDCS and rehabilitation with complex walking tasks (Sham/Complex); or sham tDCS and rehabilitation with typical walking (Sham/Typical). Outcome measures included multiple tests of walking function, executive function, and prefrontal activity during walking measured by functional near infrared spectroscopy. For the walking tests, effect sizes for Active/Complex were generally higher than for Sham/Complex. The Sham/Typical group exhibited walking test effect sizes that were often larger than either of the complex walking groups, possibly due to higher intervention step count. For the executive function tests, effect sizes were largest for the Active/Complex group. Improvements in prefrontal activity during walking were observed, as conceptualized by the Compensation Related Utilization of Neural Circuits Hypothesis. These preliminary findings support that tDCS combined with complex walking rehabilitation in older adults is feasible and may enhance both walking function and executive function.

